# *In vitro* digestion of ESC-resistant *Escherichia coli* from poultry meat and evaluation of human health risk

**DOI:** 10.3389/fmicb.2023.1050143

**Published:** 2023-02-09

**Authors:** May Linn Buberg, Yngvild Wasteson, Bjørn Arne Lindstedt, Ingun Lund Witsø

**Affiliations:** ^1^Department of Paraclinical Sciences, Faculty of Veterinary Medicine, Norwegian University of Life Sciences, Ås, Norway; ^2^Faculty of Chemistry, Biotechnology and Food Science, Norwegian University of Life Sciences, Ås, Norway

**Keywords:** virulence, ExPEC, conjugation, INFOGEST, hybrid pathogen

## Abstract

**Introduction:**

The spread of antimicrobial resistance (AMR) has become a threat against human and animal health. Third and fourth generation cephalosporins have been defined as critically important antimicrobials by The World Health Organization. Exposure to Extended spectrum cephalosporin-resistant *E. coli* may result in consumers becoming carriers if these bacteria colonize the human gut or their resistance genes spread to other bacteria in the gut microbiota. In the case that these resistant bacteria at later occasions cause disease, their resistance characteristics may lead to failure of treatment and increased mortality. We hypothesized that ESC-resistant *E. coli* from poultry can survive digestion and thereby cause infections and/or spread their respective resistance traits within the gastro-intestinal tract.

**Methods:**

In this study, a selection of 31 ESC-resistant *E. coli* isolates from retail chicken meat was exposed to a static in vitro digestion model (INFOGEST). Their survival, alteration of colonizing characteristics in addition to conjugational abilities were investigated before and after digestion. Whole genome data from all isolates were screened through a custom-made virulence database of over 1100 genes for virulence- and colonizing factors.

**Results and discussion:**

All isolates were able to survive digestion. Most of the isolates (24/31) were able to transfer their *bla*_*CMY*2_-containing plasmid to *E. coli* DH5-á, with a general decline in conjugation frequency of digested isolates compared to non-digested. Overall, the isolates showed a higher degree of cell adhesion than cell invasion, with a slight increase after digestion compared non-digested, except for three isolates that displayed a major increase of invasion. These isolates also harbored genes facilitating invasion. In the virulence-associated gene analysis two isolates were categorized as UPEC, and one isolate was considered a hybrid pathogen. Altogether the pathogenic potential of these isolates is highly dependent on the individual isolate and its characteristics. Poultry meat may represent a reservoir and be a vehicle for dissemination of potential human pathogens and resistance determinants, and the ESC-resistance may complicate treatment in the case of an infection.

## Introduction

The diverse family of *Enterobacteriaceae* includes one of the most studied microbes, *Escherichia coli*. Within *E. coli*, we find commensals, opportunistic bacteria, and pathogens. Some of the pathogenic variants cause infections in the intestinal tract (intestinal pathogenic *E. coli*, IPEC), while extraintestinal pathogenic *E. coli* (ExPEC) can survive in other tissues of the host and are associated with neonatal meningitis (NMEC) and sepsis and urinary tract infections (UPEC) among others (Riley, 2014). Several virulence genes characterizing ExPEC have been described (Pitout and Laupland, 2008; Pitout, 2012; Mellata, 2013), which include genes encoding for adhesins, invasins, toxins, and siderophores and genes related to iron metabolism (Dale and Woodford, 2015). The increasing occurrence of antimicrobial-resistant ExPEC isolates has led to prolonged hospital stays and higher mortality rates (Gastmeier et al., 2012). This rise in antimicrobial resistance (AMR) has a significant impact on human and animal health (Brinkac et al., 2017; Centers for Disease Control Prevention, 2019). Recently, hybrid pathogens have been explored (Lindstedt et al., 2018), demonstrating the plasticity of the *E. coli* pangenome (Mellata, 2013) and blurring the lines between human-made bacterial classifications. An increased understanding of the dynamic flow and transmission routes of antimicrobial-resistant bacteria (ARB) and antimicrobial resistance genes (ARG) between animal and human bacterial reservoirs and host interactions is important for developing and implementing targeted measures to further prevent AMR development (VKM, 2020).

Poultry has been described as one of the main reservoirs for extended-spectrum beta-lactamase (ESBL) producing bacteria, as well as *E. coli*, which is most closely linked to human ExPEC (Carattoli, 2008). The European Food Safety Agency (EFSA) concluded in 2011 that AMR *E. coli* isolates from humans and poultry are more frequently genetically related than antibiotic-susceptible isolates and that transmission of ESBL genes, plasmids, and clones from poultry to humans is most likely to occur through the food chain (EFSA, 2011; Manges and Johnson, 2012). In 2015, the report “Assessment of AMR in the food chains in Norway” concluded that the probabilities of human exposure to ESBL-producing *Enterobacteriaceae* and their corresponding genes, from live poultry and poultry meat, were considered non-negligible (VKM, 2015). Since then, extensive measures have been taken in the Norwegian poultry industry to limit previously discovered ARB and ARGs (Mo et al., 2014, 2021; Nortura, 2016), leading to the prevalence being substantially reduced in the last few years (NORM/NORM-VET, 2017, 2019). The European Center for Disease Prevention and Control (ECDC) published in their report from 2018/2019 that the proportion of presumptive ESBL/Ampicillinase C (AmpC) producing *E. coli* was low in the animal sector, as 14 countries reported a decrease in overall prevalence, while 11 countries reported an increase of ESBL/AmpC producing bacteria (European Food Safety Authority European Centre for Disease Prevention Control, 2021).

The most common AmpC beta-lactamase encoding gene in *E. coli* is the *bla*_CMY − 2_, which is predominantly located on plasmids (Alfei and Schito, 2022). It has been reported to occur in bacteria from both human infections and various animal and food sources with increasing prevalence (Pires et al., 2022). Some authors have assessed that ESBL-producing *Enterobacteriaceae* from the broiler production chain is a considerable public health risk due to both their virulence and resistance characteristics (Pitout and Laupland, 2008; Liebana et al., 2013; Vounba et al., 2019). At the poultry slaughterhouse, it is unavoidable that intestinal bacteria contaminate carcasses during the slaughtering process (Rouger et al., 2017; Rasschaert et al., 2020; Boubendir et al., 2021). Consequently, as chicken filets are often sold as fresh products, compromised kitchen hygiene habits may result in consumers becoming exposed to these bacteria (Bloomfield et al., 2017; Santos-Ferreira et al., 2021). However, even though the probability of exposure of consumers to ESC-resistant bacteria may be high depending on the prevalence levels in live animals, less is known about the consequences of such exposure (Buberg et al., 2021). The potentially long timespan from exposure to the development of infection makes infection routes hard to trace, and there is a need for more comprehensive genetic analysis of poultry isolates to unravel their pathogenic potential and further evaluate their role as a possible risk to human health (Leverstein-van Hall et al., 2011; Manges and Johnson, 2012; Berg et al., 2017).

To be able to determine the consequences of exposure to Extended Spectrum Cephalosporin (ESC)-resistant *E. coli* through food, further investigations of the fate of these isolates through the digestion process are needed. Their survival in humans through intake by the oral route has not yet been quantified and questions regarding survival, horizontal spread of resistance genes in the gastrointestinal tract, their interactions with the intestinal cells, and possible alterations of characteristics during the digestion process remain unanswered. Many different protocols for digestion models have been described making a comparison of studies between researchers challenging (Kong and Singh, 2010; Mulet-Cabero et al., 2019; Li et al., 2020; Mackie et al., 2020). However, in 2014, the INFOGEST network published a static *in vitro* digestion model that aimed to harmonize human-digestion conditions by being an easy and applicable model that could be compared between studies (Minekus et al., 2014; Brodkorb et al., 2019). Despite the limited use of this model for microbiological purposes, it is appropriate for the evaluation of the growth and survival of *Listeria monocytogenes* (Pettersen et al., 2019).

This study aimed to contribute to the understanding of the human health risk represented by ESC-resistant *E. coli* from the poultry food chain. We hypothesized that these bacteria would survive the human digestive process and have the potential to interact with the host and/or to transfer their resistance traits to other gastrointestinal bacteria. We addressed this hypothesis by using the above-mentioned *in vitro* digestion model for the evaluation of selected isolates' survival, conjugation abilities, and ability to adhere to and invade human colorectal cells. Furthermore, we assessed the presence of virulence factors characteristic of ExPEC through an in-depth analysis of whole genome sequence data.

## Materials and methods

### Isolates and selection

A total of 141 ESC-resistant *E. coli* was isolated from domestically produced retail chicken meat in the NORM-VET programs from 2012 to 2016 (NORM/NORM-VET, 2013, 2015, 2017). All these isolates were previously whole genome sequenced and known to carry the *bla*_CMY − 2_ gene encoding ESC resistance and have previously been included in studies by Mo et al. (2014, 2016). A selection of 31 isolates was made from this collection by including isolates from all phylogroups and the most frequent sequence types, resulting in 11 isolates from 2012, 14 isolates from 2014, and six isolates from 2016. Eighteen of the isolates were previously partly characterized by Buberg et al. (2021). An overview of the included isolates is listed in Table 1.

**Table 1:** Isolates included in the study.

**Whole ID**	**Year**	**ST**	**Phylogroup**	**Previously studied published**
2012-01-3586	2012	131	B2	Mo et al., 2016; Buberg et al., 2021
2014-01-3678	2014	117	D	Mo et al., 2016; Buberg et al., 2021
2016-22-832	2016	442	B1	Buberg et al., 2021
2014-01-5656	2014	10	A	Mo et al., 2016; Buberg et al., 2021
2014-01-7037	2014	355	B2	Mo et al., 2016; Buberg et al., 2021
2016-22-220	2016	429	B2	Buberg et al., 2021
2014-01-1336	2014	1,594	A	Mo et al., 2016; Buberg et al., 2021
2012-01-1295	2012	38	D	Mo et al., 2016; Buberg et al., 2021
2012-01-707	2012	38	D	Mo et al., 2016; Buberg et al., 2021
2014-01-3680	2014	1,158	D	Mo et al., 2016; Buberg et al., 2021
2014-01-4991	2014	57	D	Mo et al., 2016; Buberg et al., 2021
2014-01-5104	2014	115	D	Mo et al., 2016; Buberg et al., 2021
2012-01-771	2012	69	D	Mo et al., 2016; Buberg et al., 2021
2014-01-7011	2014	1,944	D	Mo et al., 2016; Buberg et al., 2021
2014-01-4267	2014	191	A	Mo et al., 2016; Buberg et al., 2021
2012-01-1292	2012	38	D	Mo et al., 2016, 2017; Buberg et al., 2020, 2021
2012-01-2798	2012	3,249	A	Mo et al., 2016, 2017; Buberg et al., 2020, 2021
2016-22-1061	2016	2,040	A	Buberg et al., 2021
2012-01-1988	2012	38	D	Mo et al., 2016
2012-01-2350	2012	38	D	Mo et al., 2016
2012-01-1659	2012	10	A	Mo et al., 2016
2012-01-5334	2012	1,594	A	Mo et al., 2016
2012-01-5997	2012	10	A	Mo et al., 2016
2014-01-14	2014	38	D	Mo et al., 2016
2014-01-1676	2014	117	D	Mo et al., 2016
2014-01-2452	2014	117	D	Mo et al., 2016
2014-01-7149	2014	10	A	Mo et al., 2016
2014-01-2454	2014	38	D	Mo et al., 2016
2016-22-75	2016	1,594	A	Unpublished
2016-22-226	2016	10	A	Unpublished
2016-22-1059	2016	2,040	A	Unpublished

Isolation and species determination is described by Mo et al. (2016).

For clarification purposes and easy reading, the four last digits of the isolate ID are used when referred to in the text.

### *In vitro* digestion model

Survival after digestion was evaluated by the static *in vitro* protocol developed by INFOGEST with minor modifications to adapt the protocol for studies of bacteria (Pettersen et al., 2019). Tests determining enzymatic activity for standardization were carried out according to the protocol before the experiment. In short, overnight cultures of all bacterial isolates in LB-broth were used in the *in vitro* digestion model. The “bacterial mixture” consisting of 0.5 ml of overnight culture in broth was added to a 5-ml Eppendorf tube (Eppendorf, Hamburg, Germany). Simulated salivary fluid (SSF) was added to obtain a 1:1 ratio. CaCl_2_(H_2_O)_2_ was added to achieve a total concentration of 1.5 mM in SSF. As the bacterial mixture did not contain starch, amylase was omitted, and the mixture was incubated at 37°C for 2 min on a hematology mixer. For the gastric step, preheated simulated gastric fluid (SGF) was added to the bacterial mixture in a ratio of 1:1. The pH was adjusted to 3.0 by adding a pre-defined volume of HCl. CaCl_2_(H_2_O)_2_ was added to a final concentration of 0.15 mM in SGF. Porcine pepsin (Sigma-Aldrich, batch no. SLCF7636) (2,000 U/ml), Rabbit Gastric extract (RGE15, Lipolytech) (60 U/mL), and water were mixed to achieve a 1 × concentration of SGF, which was then added to the mixture before incubation at 37°C for 40 min (estimated passing time for liquid boluses). For the intestinal phase, preheated simulated intestinal fluid (SIF) was added to the bacterial mixture in a ratio of 1:1. The pH was adjusted to 7.0 by adding a pre-defined volume of NaOH. A final concentration of 10 mM bile was then added to the mixture. CaCl_2_(H_2_O)_2_ was added to achieve a final concentration of 0.6 mM in SIF. Pancreatin (Sigma-Aldrich, batch no. SLCF4576) with a trypsin activity of 100 U/ml was then added to the mixture. Autoclaved ddH_2_O was added to gain a 1 × concentration of the SIF before samples were incubated at 37°C for 2 h on a hematology mixer. After incubation, the bacterial mixture was diluted and plated on selective agar plates (Müeller-Hinton agar containing 0.5 mg/L cefotaxime) and incubated for 24 h at 37°C. Colonies were counted manually and CFU/ml was determined after digestion. The number of CFU for the non-digested parallel was calculated by direct plating of overnight culture on selective agar plates. The experiments were carried out in triplicate.

### Conjugation assay

Conjugation experiments were performed in LB-broth according to Buberg et al. (2020) with minor modifications. In short, the donors (all 31 isolates individually) and recipient strain (*E. coli* DH5-α) were grown overnight in LB-broth at 37°C reaching OD600 equivalent to a McFarland standard no. 1 (^*^3E+8 bacteria/ml). A volume of 500 μL of the recipient strain culture and 10 μL of the donor strain culture (donor:recipient ratio = 1:50) were mixed in 4 mL LB-broth and incubated for 4 h at 37°C. Dilutions of each mating culture were plated on Müeller-Hinton agar plates (Sigma-Aldrich, Germany) supplemented with 20 mg/L nalidixic acid and/or 0.5 mg/L cefotaxime, and incubated for 24 and 48 h at 37°C. The mating mixture was then diluted and plated on the recipient- and transconjugant-selective plates containing nalidixic acid, or both nalidixic acid and cefotaxime, respectively. The number of transconjugants was reported for quantification and comparison between the donors. Conjugation frequency was calculated by dividing the number of transconjugants by the number of recipients in CFU/mL. For control, representative colonies from each transconjugant-selective plate were plated on bromothymol lactose blue agar (Sigma-Aldrich, Germany) to distinguish transconjugants from spontaneously mutated donors (i.e., mutated to nalidixic acid resistance). In addition, PCR analysis of transconjugants was conducted to confirm that they harbored the *bla*_CMY − 2_ gene proving conjugation.

For conjugation after digestion, the same procedure was carried out immediately after the donors had gone through the *in vitro* digestion. The experiments were carried out in triplicate.

### Adhesion and invasion assay

The ability to adhere to and invade eukaryotic cells was tested in HT-29 cells (RRID: CVCL_0320) grown at 37°C (Ammerman et al., 2008) between passages 10 and 25. The cells were grown to 80% confluence, and 200 μL of cells in fresh McCoy medium (Merck, USA) with 10% Fetal Bovine Serum (GibcoTM 10270106) (referred to as McCoy+) were transferred to a microtiter plate (Corning, Costar, Fischer Scientific, USA). Plates were incubated overnight for establishing cell attachment. Cell concentration for the experiments ranged between 3 and 6 E+06 cells/ml (>90% living cells) counted with Bio-Rad TC20. Overnight culture and digested culture (digested and non-digested) of bacteria were diluted in the ratio of 1:100 in fresh LB-broth. One mL was centrifuged at 2,000 rpm for 5 min and the bacterial pellet was resuspended in 500 μL fresh McCoy+ medium without antibiotics. The bacterial suspension was diluted in the ratio of 1:100 and 50 μL of bacterial suspension was added to each well (equivalent to MOI 30:1). Plates were centrifuged at 1,000 rpm for 2 min to increase contact between bacteria and cells, and incubated for 2 h at 37°C. To assess total cell association (number of adhering and invading bacteria), the cells were washed three times with PBS (1 × ) to remove non-adherent bacteria and lysed with 30 μL 1% Triton X for 10 min. The lysates were serially diluted in PBS (1 × ) and plated on selective agar as previously described. To assess bacterial invasion, 200 μL of fresh medium with antibiotics (0.1 mg/ml gentamicin and 20 mg/mL nalidixic acid) was added to the cells before incubation at 37°C for 2 h to kill adherent bacteria. The cells were washed, lysed, and plated as described previously. Adherence was calculated by subtracting numbers from the cell invasion from the total amount of cell-associated bacteria. Digested and non-digested were compared, and experiments were carried out in triplicate.

### Detection of virulence genes

An extended virulence-associated gene analysis was carried out. The sequences were scanned (i.e., BLAST search) against a custom database previously used for characterizing environmental isolates (Finton et al., 2020) now expanded to include 1,191 genes/gene variants or genetic markers. The database contains genes related to both ExPEC and IPEC as well as loci suspected to contribute to virulence, e.g., the ETT2 locus. Only matches with 95% or more nucleotide identity with 60% or more query coverage were included in the results.

## Results

### *In vitro* digestion

**Figure 1:**
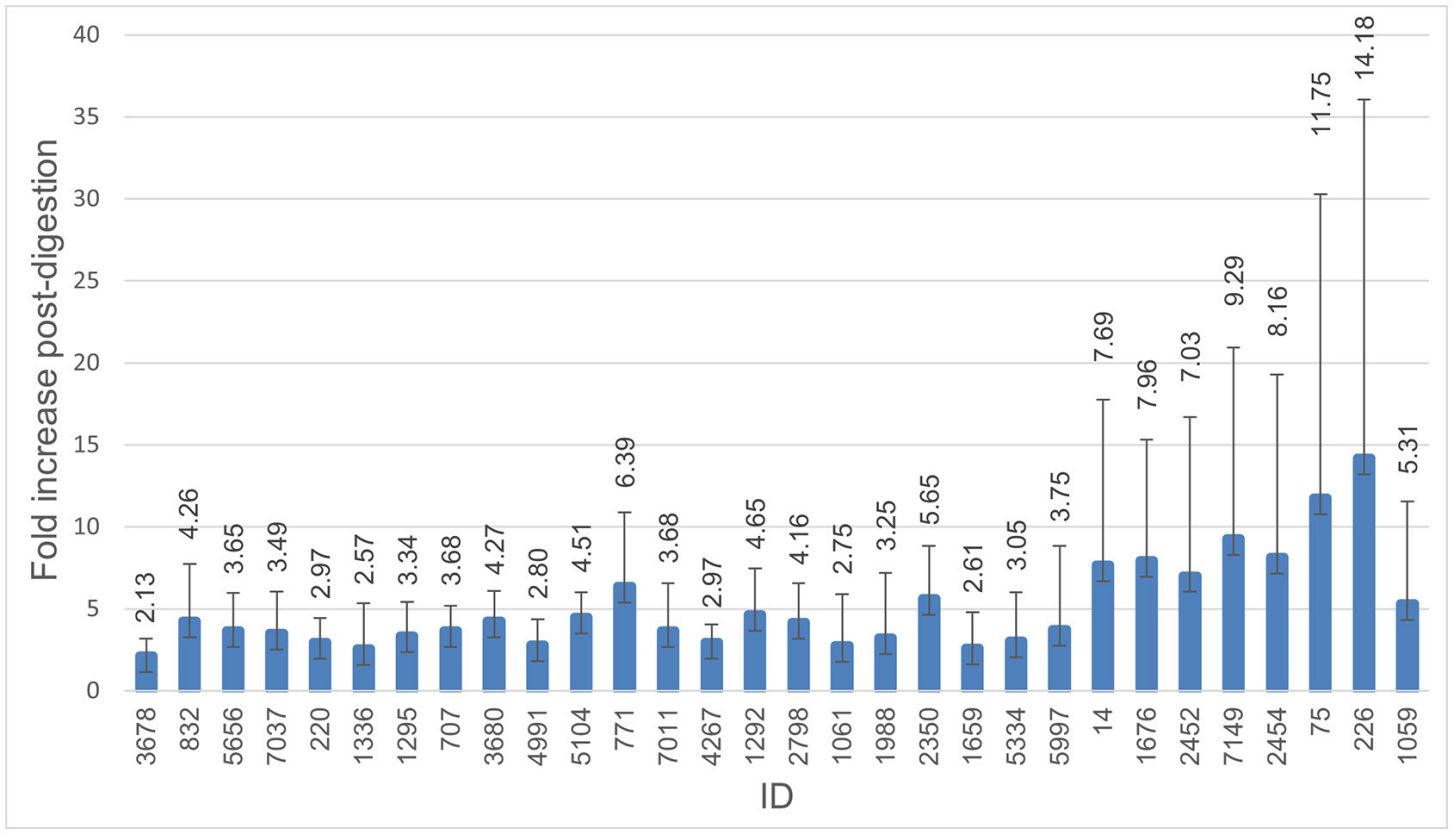
Survival through digestion. Histogram demonstrating fold-increase in CFU/ml after digestion compared to non-digested. The error bar represents the standard deviation. All isolates were able to survive digestion, and all were able to continue growth during the digestion process. Isolate 3,586 was excluded from this figure due to a failure of growth in the non-digested control.

All isolates were able to survive digestion in the INFOGEST *in vitro* digestion model. The numbers of the colony-forming units (CFU/mL) for digested and non-digested isolates are shown in Figure 1. The number of bacteria after digestion varied between isolates and was highly increased for some isolates and replicates. Mean CFU/mL for non-digested isolates was 4.91E+08, compared to 1.82E+09 for digested isolates. All isolates showed a 1-fold increase or higher in CFU/ml, with an average of 5.31-fold increase. More than a 4-fold increase was seen in 15 of the 31 isolates, with isolate 226 displaying the highest increase in the number of CFU/ml after digestion at a 14.18-fold increase.

### Conjugation

**Figure 2:**
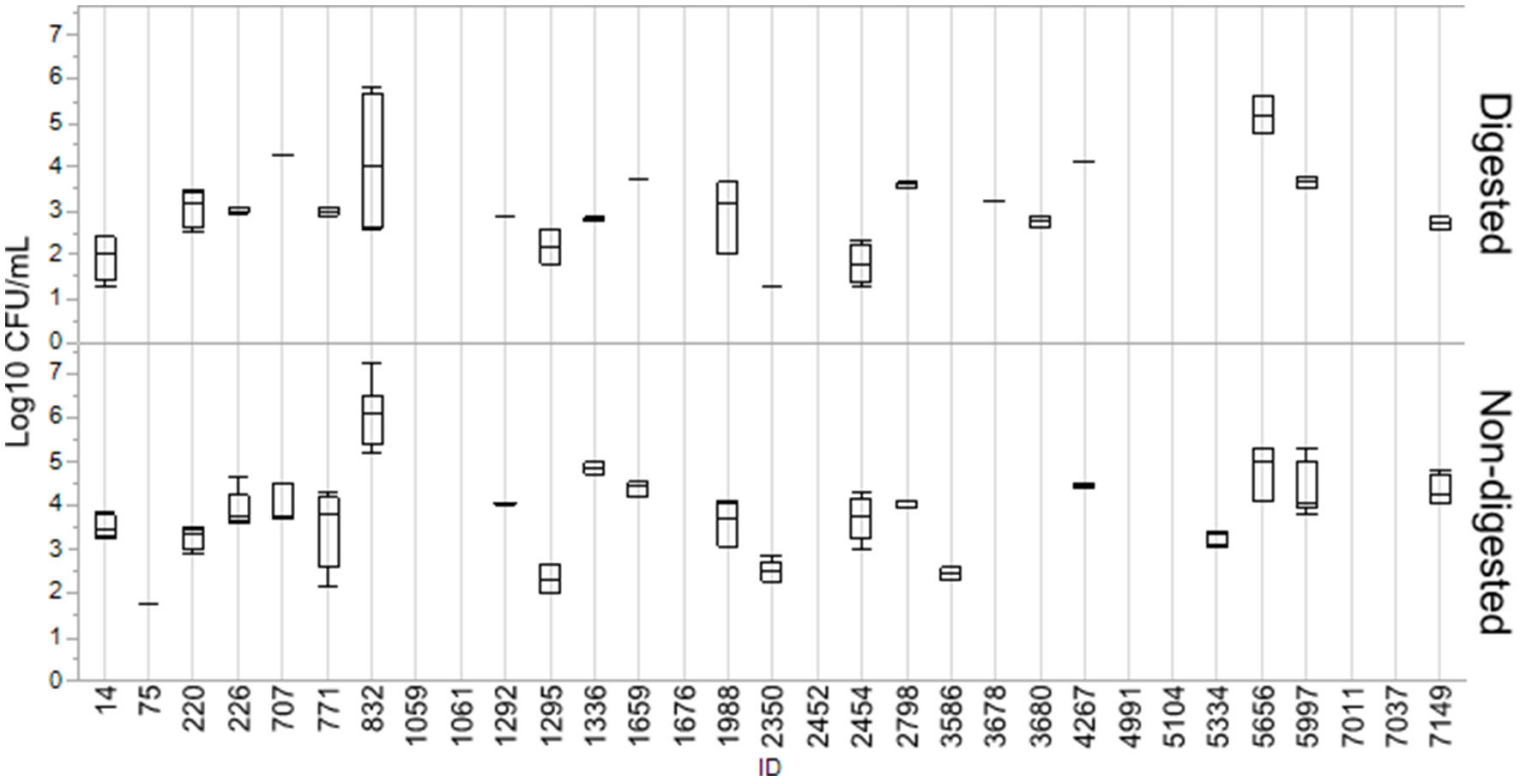
Log10 CFU/ml of transconjugants for digested and non-digested isolates. Boxes represent the quartiles and the median value, with included confidence intervals as the standard error of mean indicated by whiskers. Values are given in the number of Log10 CFU/ml. No whiskers are visible for isolates where the outer quartile is equal to the minimum or maximum value. For isolates where no transfer was detected, no box is present.

Numbers of CFU/ml of transconjugants digested and non-digested for each donor are illustrated in Figure 2. Table 2 gives an overview of the sample population and highlights the variation of conjugation frequencies before and after digestion. Conjugation frequencies are available in the Supplementary material.

**Table 2:** Ability to conjugate in liquid broth.

**Conjugation: digestion**	**Total**	**Conjugation after digestion**	**NTD after digestion**
Total	100% (31)	64.51% (20/31)	35.49% (11/31)
Conjugation non-digested	67.74% (21/31)	85.71% (18/21)	14.28% (3/21)
NTD non-digested	32.25% (10/31)	20.00% (2/10)	80.00% (8/10)

NTD, no transfer detected.

Binary distribution of the number of isolates able to successfully transfer *bla*_CMY − 2_ to *E. coli* DH5-α after digestion compared to non-digested. If no confirmable transconjugants were present on the transconjugant-selective plates, it was considered NTD.

In general, most isolates that were able to transfer their *bla*_CMY − 2_ gene displayed a decrease in conjugation frequencies after digestion. Out of the 31 isolates, 24 were able to transfer *bla*_CMY − 2_ before or after digestion, and 19 consequently before and after. Eight isolates (7037, 4991, 5104, 2452, 1059, 1061, 1676, and 7011) were not able to conjugate at all. Conjugation frequency (the number of CFU/mL of transconjugants divided by the number of CFU/mL recipients) for each digested and non-digested isolate is reported in Supplementary Table S1. The mean conjugation frequency for non-digested was 2.04E−03, compared to digested which was 3.60E−04. Isolates 3687 and 3680 were only able to conjugate after being exposed to digestion. While isolates 0075, 3586 and 5334 were only able to conjugate before being digested, and not after. Figures are made in JMP Pro 16.0.0.

### Adhesion and invasion assay

**Figure 3:**
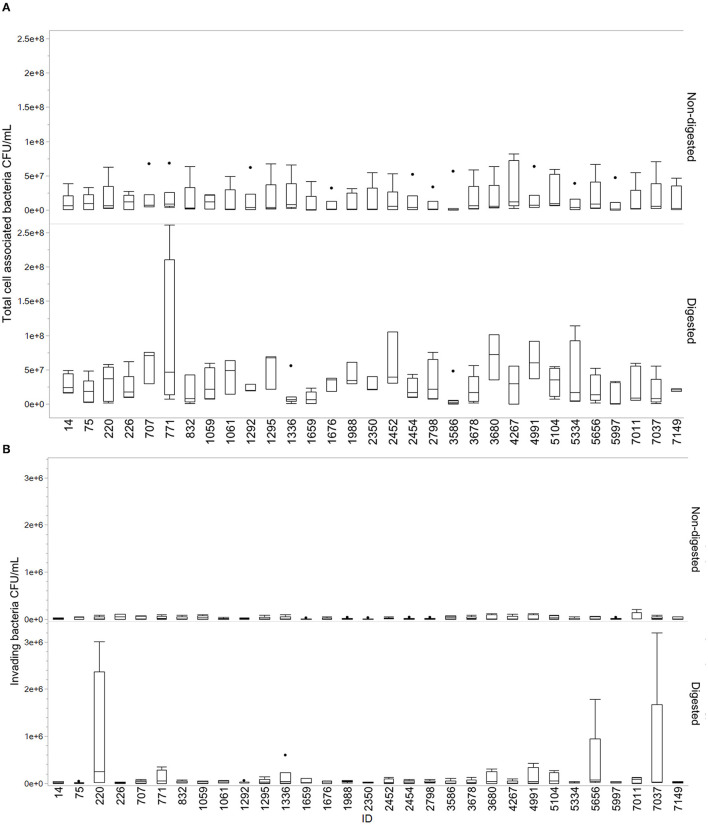
Total cell-associated bacteria and invading bacteria for digested and non-digested. Boxes represent the quartiles and the median value, with included confidence intervals indicated by whiskers. Values are given in number of CFU/mL. The dots represent outliers in the data. **(A)** Total cell-associated bacteria in Q18 CFU/mL for digested and non-digested. **(B)** Number of invading bacteria in CFU/mL for digested and non-digested.

Overall, the isolates displayed a greater ability to cell adhesion compared to cell invasion. Most isolates showed an increase in total cell association after digestion compared to non-digested isolates. Results from this assay are demonstrated in Figure 3. The mean of cell adhesion for non-digested was 1.55E+07 compared to digested at 3.36E+07. For invasion, the mean for non-digested was 2.10E+04 and digested was 1.03E+05. Some variability was observed between the different replicates. Isolates 1336, 1659, 3586, and 4267 were the only isolates for which a decrease in adhesion was observed after digestion. For invasion, especially isolates 220, 5656, and 7037 stood out with highly increased invasion numbers after digestion compared to non-digested. Figures are made in JMP Pro 16.0.0.

### Virulence-associated gene analysis

**Figure 4:**
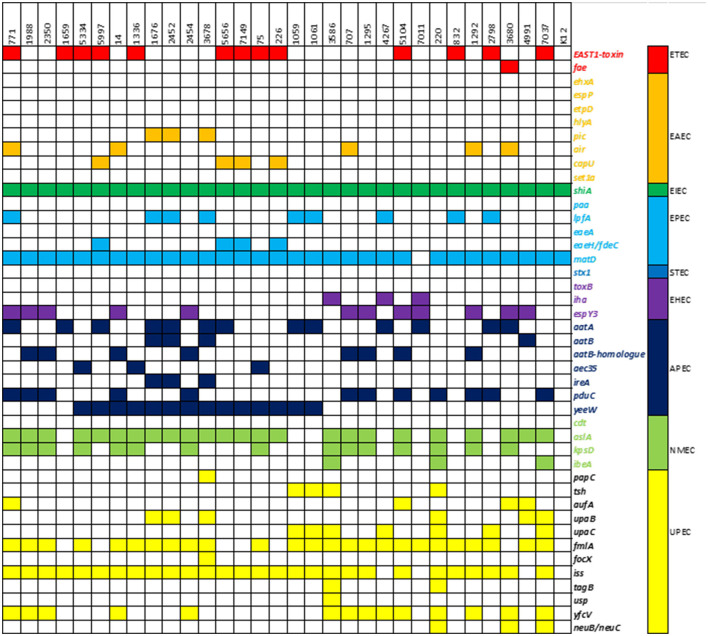
Heatmap of virulence-associated genes: presence of a selection of 41 virulence-associated genes and their respective pathogroups in the study population.

The presence of virulence-associated genes was compared to a reference *E. coli* K12 strain. Out of 1,191 genes in the database, our isolates had 123–193 (mean 161) different genes, compared to 107 in *E. coli* K12. Fifty-one genes were found in all isolates, including the control strain, and were considered core genes of the *E. coli* genome. An overview of 41 relevant virulence genes is presented in Figure 4, while a more detailed overview is available in the Supplementary material. According to the UPEC definition by Spurbeck et al. (2012), two of the isolates (220 and 7037) were predicted to be human UPEC isolates by containing the genes *chuA, fyuA*, and *yfcV* while being negative for *vat*. These two isolates were also both positive for other well-documented ExPEC/UPEC-associated genes, e.g., *papC, upaC, ibeA, irp1, irp2*, and *kpsMII*. In addition, isolate 7037, as the only isolate in our collection, contained the *gimB*-genomic island, which is associated with the invasion process of the host cells, particularly in NMEC strains, but is also found in APEC strains (Ewers et al., 2007). Isolate 220 held the *tagB/tagC* (Pokharel et al., 2020), and three isolates (220, 3680, and 7037) were considered K1-isolates as they held *neuB/neuC*, which is related to ExPEC virulence. The ability to acquire iron is related to pathogenicity. The majority of isolates contained iron acquisition loci in which *chuA, sit*, and the aerobactin loci were prominent. None of the isolates contained the ExPEC-related toxin genes *pic, sat, vat, hlyA*, or *cnf* . Genes associated with IPEC strains were detected as isolate 3680 contained *ehaA* (adhesion) and *espY3* non-LEE effector gene associated with EHEC/EPEC and *fae* (F4 fimbriae) genes associated with ETEC (Larzábal et al., 2018), and can thus be considered a hybrid pathogen.

## Discussion

This study aimed to assess potential health risks represented by ESC-resistant *E. coli* from poultry meat. We showed that the selected isolates were able to survive and multiply during gastrointestinal digestion *in vitro* and that they were able to adhere to and invade human colorectal cells after digestion. In addition, we demonstrated that the stress of being digested changed the conjugation frequency of the *bla*_CMY − 2_-containing plasmid harbored by these bacteria. The presence of virulence-associated genes was evaluated for further determination of the pathogenic potential of the selected isolates. In general, the isolates showed a large variety of gene content despite being of the same origin and carrying the same AmpC phenotype and *bla*_CMY − 2_ resistance gene. The impact on human health due to exposure to ESC-resistant *E. coli* from poultry meat therefore strongly depends on the individual bacterial isolate being involved.

It was an expected finding that all the investigated isolates were able to survive digestion and displayed an increase in CFU/ml after digestion (Figure 1), as the fecal-oral pathway for infection is common for *Enterobacteriaceae* (Tenaillon et al., 2010). The bacteria were not too hampered by the low pH in the gastric step and were able to replicate despite the limited nutrients available in the simulated gastrointestinal fluids throughout digestion. One study investigated the survival of acid-sensitive bacteria and showed that survival increased in the presence of solid foods (Waterman and Small, 1998). The survival of *E. coli* is therefore expected to increase further with more nutrients available in the form of a meal. If, for example, consumers get exposed to ESC-resistant *E. coli*, these bacteria might therefore reach, encounter, and subsequently interact with the resident microflora.

The current study assessed the conjugational abilities of the *bla*_CMY − 2_ gene in a liquid broth (Table 2). The overall tendency when analyzing the conjugation data was that the frequency of spread of *bla*_CMY − 2_ decreased after digestion (Figure 2). Several studies have suggested that stress may enhance the further spread of resistance or virulence plasmids by triggering the SOS response (Baharoglu et al., 2010; Pribis et al., 2019). As the process of digestion includes an extreme change in pH, in addition to digestive enzymes, it can be considered a stressful procedure for the bacteria. We hypothesized that this stress would increase the spread of resistance genes and increase the bacteria's ability to adhere to and invade gastrointestinal cell lines, making colonizing of the gut more likely. Some of the isolates did not transfer the *bla*_CMY − 2_ gene to the recipient *E. coli*, as demonstrated in Table 2. The reason for this conjugation failure was not further investigated. Another interesting observation in this study is the large variation in the isolates' ability to transfer the *bla*_CMY − 2_ gene with changing conditions, demonstrating that despite their similarities, they likely have a variable ability to adapt to the changing environment of the gastrointestinal tract. Replicon typing of the respective isolates has been previously performed by Mo et al. (2016). In brief, all isolates included in this study hold an IncK plasmid, with exception of 1061 and 2798 which hold only an IncI1 plasmid. Isolate 1336 holds both IncK and IncI1, while isolate 1295 has IncK, IncFII, and IncFIB. No correlation between conjugation and replicon type was seen in this study and thus has not been investigated further.

Adherence and invasion are important characteristics of pathogen–host interactions (Kalita et al., 2014; Desvaux et al., 2020). We observed a change in the ability of cell interaction after digestion compared to non-digested (Figure 3). The results varied between isolates, which may demonstrate that single isolates display a higher probability than others to establish themselves in the gastrointestinal tract. The act of colonizing the intestinal tract is not dependent on individual bacterial characteristics alone but is a complex dynamic involving host factors, the residing microbiota, the nutrients available, and qualities of the colonizing strain (Srikanth and McCormick, 2008; Tenaillon et al., 2010; Richter et al., 2018). Interestingly, isolate 7037 displayed an extremely high invasion rate in one of the replicates, which contributed to the increase in the mean and spread of data (Figure 3). This isolate holds the GimB operon, which is important for NMEC pathogenicity, in addition to *ibeA*, which is related to invasion (Ewers et al., 2007). Only two other isolates contained the *ibeA* gene, isolate 3586 and 220, the latter also displaying an increased cell invasion after digestion. When assessing the ability to adhere to and invade human colorectal cells, the current study found an overall increasing trend of cell association after the digestion procedure.

To our knowledge, the survival of ESC-resistant *E. coli* through an *in vitro* digestion model has not been previously studied. Our findings are in concurrence with a study using an *in situ* model that demonstrated the survival and colonizing abilities of an ESBL-resistant *E. coli* strain from poultry. However, this study only focused on the latter steps of digestion (cecum and colon) and was only performed for a single isolate (Smet et al., 2011). *In vivo* digestion models are thought to resemble the most life-like conditions but raise ethical questions as they require living animals or human volunteers to carry them out. They are in addition time-consuming, expensive, and require specialized facilities. A large variety of *in vitro* digestion models have been established as good alternatives to *in vivo* models. The dynamic models are the ones that simulate the most accurate digestion; however, these are still expensive, and the comparison of results between different laboratories has proven difficult. Due to the recent harmonization of the INFOGEST static *in vitro* digestion model, it is now possible to standardize research regarding digestibility across laboratories. The current model has been adjusted with minor modifications to fit microbiological studies such as the survival of bacteria as used in this study. A limitation of this study is that a static *in vitro* assay does not exactly replicate the conditions in the gastrointestinal tract. However, this model has been documented to be physiologically comparable to *in vivo* porcine digestion of skim milk powder (Egger et al., 2017). In addition, dietary and genetic host factors that may affect the individual host-bacteria interactions in the gastrointestinal tract have not been considered in the current study.

Determination of different pathovars of *E. coli* based on the analysis of their virulence gene content is highly dependent on the database used, as there may be individual differences in which genes are included in the defining criteria. This study used a custom-made database containing over 1,191 genes to increase the coverage of the number of VAGs and compared the results to a common K12 *E. coli* strain. Based on the genotypic results, isolates 220 and 7037 should be classified as human UPEC. A previous analysis of a subgroup of the isolates included in this study concluded that the risk of developing UTI upon exposure to ESC-resistant *E. coli* from poultry was limited. Nevertheless, with this expanded knowledge by applying a wider VAGs search, the risk of causing disease appears to be higher than first anticipated (Buberg et al., 2021). Except for isolates 220 and 7037, there were very few virulence traits connected to known human pathogenic variants of *E. coli*. The strain 3680 contained some genes (e.g., *ehaA* and *espY3*) associated with both enterohemorrhagic/enteropathogenic *E. coli* (EHEC/EPEC) and Shigatoxin-producing *E. coli* (STEC) (Figure 4). However, the lack of specific toxins makes it questionable whether this isolate should be classified as an IPEC strain.

In summary, the current study aimed to evaluate the consequences of consumer exposure to ESC-resistant *E. coli* using a static *in vitro* digestion model and evaluated conjugation and cell adhesion and invasion for digested and non-digested isolates, in addition to performing an in-depth VAG analysis to evaluate ExPEC potential. We conclude that the pathogenic potential is highly dependent on the characteristics of the individual isolates. The isolates 7037, 220, and 3680 contained genes and characteristics classifying them as ExPEC. As they additionally encode and express ESC resistance, they may complicate treatment in the case they cause disease in a human host. This study demonstrates that poultry meat may, although to a limited extent, represent a reservoir and be a vehicle for the dissemination of potential human pathogens and plasmid-borne resistance determinants such as *bla*_CMY − 2_.

## Data availability statement

The raw data supporting the conclusions of this article will be made available by the authors, without undue reservation.

## Author contributions

MB planned, carried out the experiment, and interpretated and analyzed the results. MB wrote the manuscript with support from YW, BL, and IW. BL performed the VGA analysis. YW and IW supervised the project. All authors provided critical feedback and helped shape the research, analysis, and manuscript. All authors contributed to the article and approved the submitted version.
