# Supplementary material for: In vitro digestion of ESC-resistant Escherichia coli from poultry meat and evaluation of human health risk
Source: Front Microbiol. 2023 Feb 9;14:1050143. doi: 10.3389/fmicb.2023.1050143 (PMC9947789; doi:10.3389/fmicb.2023.1050143)
Supplement: Supplementary file 1 [file Table_1.DOCX]

Table S1: Conjugation frequencies before and after digestion. Ratio = Number of transconjugants (CFU/mL) divided by number of recipients. Numbers are based on an average of three replicates. NTD = No transfer detected. *Isolate 1295 mutated in 2/3 after digestion experiments, and true transconjugants were therefor only observed in a single replicate, hence no SD.

|  | Non-digested | | Digested | |
| --- | --- | --- | --- | --- |
| **ID** | Ratio | SD | Ratio | SD |
| 3586 | 6.17E-06 | 1.07E-05 | NTD | 0.00E+00 |
| 3678 | NTD | 0.00E+00 | 1.38E-04 | 2.39E-04 |
| 832 | 3.59E-02 | 4.42E-02 | 4.35E-03 | 7.29E-03 |
| 5656 | 1.30E-03 | 2.05E-03 | 1.52E-03 | 3.23E-03 |
| *7037* | NTD | 0.00E+00 | NTD | 0.00E+00 |
| 220 | 2.86E-05 | 1.01E-05 | 7.97E-05 | 7.22E-05 |
| 1336 | 1.11E-03 | 7.26E-04 | 5.00E-05 | 1.06E-04 |
| 1295* | 4.90E-06 | 6.42E-06 | *2.24E-06* | 0.00E+00 |
| 707 | 3.05E-04 | 4.14E-04 | 1.92E-04 | 4.08E-04 |
| 3680 | NTD | 0.00E+00 | 7.82E-05 | 1.66E-04 |
| *4991* | NTD | 0.00E+00 | NTD | 0.00E+00 |
| *5104* | NTD | 0.00E+00 | NTD | 0.00E+00 |
| 771 | 8.81E-05 | 8.81E-05 | 8.64E-06 | 1.50E-05 |
| 7011 | NTD | 0.00E+00 | NTD | 0.00E+00 |
| 4267 | 8.38E-04 | 8.80E-04 | 2.18E-04 | 3.77E-04 |
| 1292 | 2.52E-04 | 2.61E-04 | 2.74E-05 | 4.75E-05 |
| 2798 | 1.54E-04 | 3.28E-04 | 1.05E-04 | 1.81E-04 |
| 1061 | NTD | 0.00E+00 | NTD | 0.00E+00 |
| 1988 | 1.22E-04 | 7.66E-05 | 8.00E-05 | 1.09E-04 |
| 2350 | 4.99E-05 | 7.40E-05 | 1.59E-06 | 2.75E-06 |
| 1659 | 3.59E-04 | 1.80E-04 | 2.01E-04 | 3.48E-04 |
| 5334 | 4.02E-05 | 2.32E-05 | NTD | 0.00E+00 |
| 5997 | 1.03E-03 | 9.70E-04 | 9.96E-05 | 1.72E-04 |
| 14 | 5.70E-05 | 4.23E-05 | 1.33E-05 | 8.12E-06 |
| 1676 | NTD | 0.00E+00 | NTD | 0.00E+00 |
| 2452 | NTD | 0.00E+00 | NTD | 0.00E+00 |
| 7149 | 6.75E-04 | 5.52E-04 | 1.09E-05 | 1.88E-05 |
| 2454 | 2.22E-04 | 1.16E-04 | 1.29E-05 | 1.41E-05 |
| 75 | 2.05E-07 | 4.35E-07 | NTD | 0.00E+00 |
| 226 | 3.07E-04 | 3.55E-04 | 2.10E-05 | 3.64E-05 |
| 1059 | NTD | 0.00E+00 | NTD | 0.00E+00 |
